# Simulated Body Fluids for Dental Implant Corrosion: A Practical Guide

**DOI:** 10.3390/dj14050292

**Published:** 2026-05-12

**Authors:** Aydin Bordbar-Khiabani

**Affiliations:** VTT Technical Research Centre of Finland Ltd., Tekniikantie 21, FI-02150 Espoo, Finland; aydin.bordbar-khiabani@vtt.fi or aidinbordbar@gmail.com

**Keywords:** dental implants, electrochemical corrosion, artificial saliva, physiological simulants, inflammatory conditions, microbiological corrosion

## Abstract

**Background/Objectives**: Electrolytes used in in vitro corrosion testing critically determine the behavior inferred for metallic dental implants, yet formulations and their justifications are inconsistently reported across the literature. This review compiles and compares electrolytes employed to simulate the oral cavity and the bone–implant interface, linking their chemical composition to the corrosion mechanisms they target. **Methods**: This structured narrative review synthesized peer-reviewed literature on simulated electrolytes used for in vitro corrosion testing of metallic dental implants and implant-related alloys. Literature was identified using database searches and targeted reference screening, with emphasis on artificial saliva formulations, physiological simulated fluids, challenge chemistries, protein-containing media, hydrodynamic conditions, and microbiological models. Relevant formulations were standardized to grams per liter and grouped according to application domain and targeted corrosion mechanisms. **Results**: The analysis maps electrolyte selection to corresponding corrosion modes, including uniform dissolution, pitting, crevice, galvanic, and microbiologically influenced corrosion. Consolidated composition tables highlight how pH, halide concentration, calcium–phosphate balance, proteins, gas control, and flow conditions modify passive-film stability and metal-ion release. Dental-specific gaps are identified, notably the lack of a standardized fluoride–pH matrix and limited guidance for microbiome-integrated assays. **Conclusions**: Aligning electrolyte formulations with the research question enhances reproducibility and mechanistic interpretation. However, current in vitro corrosion data should be interpreted cautiously because quantitative links between simulated-fluid testing and clinical outcomes such as peri-implantitis, peri-implant bone loss, and implant failure remain insufficiently established. The adoption of shared reporting standards, dynamic programmable chemistries, and interoperable datasets may improve the translational value of future corrosion studies.

## 1. Introduction

**Figure 1:**
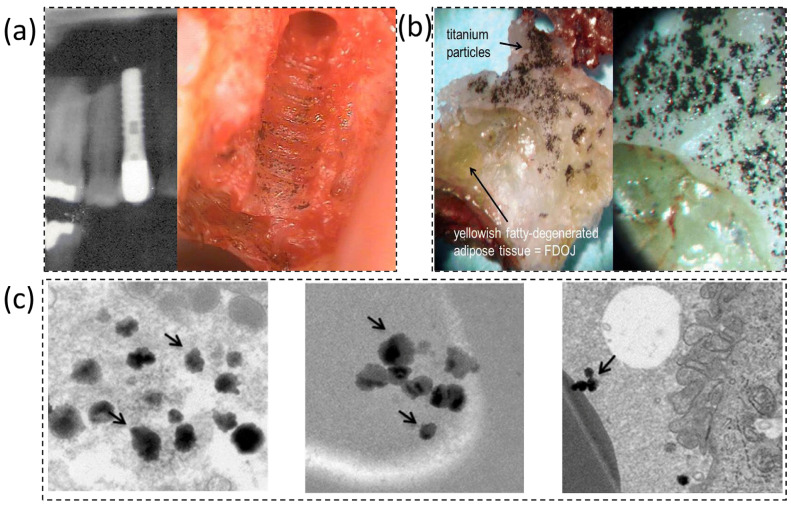
Evidence of titanium release in peri-implant sites. (**a**) Pre-removal radiograph of a titanium dental implant; the adjacent alveolar bone shows speckled radiopacities consistent with precipitated titanium particles. (**b**) Macroscopic view of a jawbone fragment with metallic debris visible to the naked eye (titanium particles), reproduced and adapted from [7] under Creative Commons Attribution 4.0 International License (CC BY 4.0). (**c**) TEM micrographs of peri-implant tissue showing aggregates of Ti-based nanoparticles (arrows), reproduced and adapted from [8] under Creative Commons Attribution 4.0 International License (CC BY 4.0).

Dental implants function within a complex biochemical environment that encompasses both the oral cavity and the surrounding bone tissue, exposing them to distinct yet interacting conditions [1]. In the oral cavity, the exposed implant surfaces, such as the abutment and transmucosal region, are continually bathed in saliva containing various ions, enzymes, and microbial metabolites [2]. Salivary pH fluctuates between acidic and near-neutral values due to dietary acids, bacterial biofilm activity, or oral hygiene products, while components such as fluoride from toothpaste or mouth rinses further modify the local chemistry [3]. In contrast, the portion of the implant embedded within bone is in contact with extracellular fluid resembling blood plasma, which provides an ionic environment rich in calcium, phosphate, and proteins [4]. During the initial healing phase (osseointegration), the peri-implant milieu includes blood clot and wound exudates; in cases of peri-implant inflammation, it becomes infiltrated with immune cells and inflammatory mediators [5]. Corrosion processes may occur in both environments, ultimately contributing to adverse outcomes such as peri-implant tissue inflammation, release of metal ions or particles, and degradation of the implant’s structural integrity [6]. The presence of corrosion by-products around implants has indeed been associated with peri-implantitis and bone resorption, potentially compromising implant stability and longevity [7]. As illustrated in Figure 1, representative radiographic, macroscopic, and transmission electron microscopy (TEM) images demonstrate titanium particle deposition in peri-implant tissues [7].

At the material level, titanium and its alloys (e.g., Ti-6Al-4V and titanium–zirconium alloys) derive their corrosion resistance from the spontaneous formation of a nanometer-thin oxide layer (primarily TiO_2_) on the surface [8]. This passive film provides effective protection in neutral physiological environments; however, it can be compromised under certain conditions prevalent in the oral cavity [9]. For instance, low pH levels or the presence of aggressive species such as chloride, proteins, and lactate can disrupt or dissolve the TiO_2_ layer, leading to accelerated corrosion of the underlying metal [10]. Once the passive film is breached, the implant surface becomes susceptible to electrochemical attack, resulting in pitting or generalized corrosion and the subsequent release of titanium ions into the surrounding tissues [11].

Another significant degradation mechanism in dental implants is galvanic and crevice corrosion, which frequently occur at the junctions between different components of the implant system. When two dissimilar metals (for example, a titanium implant and a cobalt–chromium alloy abutment screw) are in direct contact within the conductive medium of saliva or body fluid, a galvanic couple may form, promoting corrosion of the more anodic metal [12]. In addition, the implant–abutment interface inherently contains a microgap capable of trapping fluid and bacteria; this crevice environment tends to become acidic and oxygen-depleted over time [13]. Such conditions are conducive to crevice corrosion, resulting in localized attack on metal surfaces concealed within the interior of the connection. Evidence from retrieved implants has shown clear signs of corrosion at internal implant–abutment connections, including discoloration, pitting, and surface cracking, attributable to these combined galvanic and crevice processes [7].

Besides purely chemical or galvanic processes, combined mechanical and biological factors also contribute to implant corrosion. The synergy between wear and corrosion, often referred to as tribocorrosion, arises from micro-movements at contacting surfaces under functional loads (for example, at the implant–abutment joint during chewing) [14]. Repeated fretting motion can mechanically disrupt the oxide film, and when this occurs in a corrosive environment, metal release is accelerated [15]. At the same time, microbial biofilms on implants produce organic acids and oxidizing agents such as hydrogen peroxide that locally lower pH and attack the titanium surface, a phenomenon known as microbiologically influenced corrosion (MIC) [16]. The presence of a biofilm not only introduces these chemical aggressors but also alters frictional conditions, intensifying the wear–corrosion cycle at the implant surface [17]. Consequently, tribocorrosion and MIC often act synergistically, leading to greater accumulation of titanium debris and ions in the peri-implant tissues [18].

In light of these corrosion challenges, in vitro studies are widely employed to investigate implant materials under simulated conditions [19,20]. A crucial consideration in such experiments is the choice of electrolyte solution, as it largely determines the chemical environment driving corrosion reactions. Subtle differences in electrolyte composition, including pH, ionic strength, halide concentration, the presence of calcium and phosphate ions, and the buffering system, can lead to markedly different corrosion behaviors for the same implant material [21,22]. For example, artificial saliva formulations used to mimic the oral cavity often contain chloride ions and organic components (and sometimes fluoride), whereas simulated body fluids or cell culture media include calcium, phosphate, and proteins to replicate the peri-implant bone environment [23,24]. Factors such as dissolved oxygen levels, flow agitation, and the presence of organic ligands further influence the stability and reformation of the TiO_2_ layer during testing [25].

Because there is currently no single standardized testing solution for implant corrosion, researchers have reported widely varying results across studies, making direct comparisons difficult. The aim of this review is therefore to collect, categorize, and analyze the electrolyte formulations employed in dental implant corrosion studies, highlighting their composition, rationale, and clinical relevance. To the best of our knowledge, no prior review has comprehensively compiled these formulations in a single work, and this synthesis provides researchers with a practical reference for selecting appropriate test media in future investigations.

## 2. Review Methodology

This article was designed as a structured narrative review rather than a systematic review or meta-analysis. The objective was to identify, categorize, and discuss simulated electrolytes used in in vitro corrosion studies of metallic dental implants and implant-related alloys, with emphasis on the relationship between solution chemistry and corrosion mechanisms.

A literature search was conducted using PubMed/MEDLINE, Scopus, Web of Science, and Google Scholar. The search covered studies published up to April 2026. The following keywords and combinations were used: “dental implant corrosion”, “titanium corrosion”, “Ti-6Al-4V corrosion”, “cobalt chromium dental alloy corrosion”, “stainless steel dental corrosion”, “artificial saliva”, “simulated saliva”, “simulated body fluid”, “SBF”, “PBS”, “Hank’s balanced salt solution”, “HBSS”, “Ringer’s solution”, “synthetic blood plasma”, “fluoride corrosion”, “low pH”, “peri-implantitis”, “inflammatory condition”, “hydrogen peroxide”, “reactive oxygen species”, “microbiologically influenced corrosion”, “biofilm corrosion”, and “tribocorrosion”.

Studies were considered relevant when they:

(i) investigated corrosion, ion release, passive-film stability, tribocorrosion, or microbiologically influenced corrosion of dental implant materials or related biomedical alloys;
(ii) used artificial saliva, physiological simulated fluids, cell culture media, or challenge electrolytes relevant to oral or peri-implant environments;
(iii) reported electrolyte composition, pH, additives, or testing conditions sufficiently to support mechanistic interpretation; or
(iv) provided standard formulations or methodological guidance relevant to corrosion testing.

Studies were excluded when they were unrelated to dental or implant materials, did not involve metallic biomaterials, did not describe the electrolyte or exposure medium, or focused solely on clinical outcomes without relevance to corrosion mechanisms or simulated environments. Additional references were identified by manual screening of reference lists from relevant reviews, standards, and highly cited experimental studies.

Because this work was designed as a structured narrative review, no formal risk-of-bias assessment, evidence-quality grading, or meta-analysis was performed. The included studies were evaluated descriptively according to electrolyte composition, chemical complexity, simulated clinical environment, corrosion mechanism, and relevance to dental implant materials. This approach supports a practical comparison of test media and corrosion mechanisms but limits the ability to draw quantitative conclusions regarding clinical outcomes such as peri-implantitis, peri-implant bone loss, or implant failure. Reported formulations were converted, where possible, to grams per liter to facilitate comparison across studies.

## 3. Electrolyte Selection for Oral and Physiological Simulations

In vitro corrosion tests must use electrolytes that closely replicate the oral cavity and bone–implant interface conditions, with biological fluid composition serving as the basis for testing (Table 1). For supragingival and subgingival environments, artificial saliva formulations that reflect human saliva chemistry are commonly employed [26]. These solutions can be adjusted to simulate specific challenges, such as acidic pH (e.g., Fusayama–Meyer solution) to mimic dietary acids or the addition of sodium fluoride to represent exposure from dental products. Organic components like urea, mucin, and albumin are also included to reproduce the influence of salivary proteins and macromolecules on corrosion and wear [27]. Such modifications allow investigation of tribocorrosion under acidic conditions, chloride-induced pitting from low pH or saline foods, and fluoride-assisted passive film degradation. Aligning the saliva simulant with targeted oral conditions enhances the clinical relevance of corrosion testing during food intake, oral hygiene practices, or bacterial activity [28].

At the bone–implant interface, corrosion testing typically uses buffered physiological solutions such as PBS to maintain near-physiological pH and ionic strength. More complex media, including Ringer’s solution, HBSS, and SBF, incorporate additional ions (e.g., Ca and P) to better replicate bone extracellular fluid and enable apatite-like calcium phosphate formation on titanium alloys, which is an important consideration when evaluating bioactive coatings [29]. Cell culture media such as DMEM with serum further enhance realism by introducing proteins and metabolites that influence long-term corrosion behavior [30].

Selecting an appropriate electrolyte is critical, as oversimplified solutions (e.g., dilute NaCl) may neglect key constituents such as sulfur-containing amino acids or proteins, which can either accelerate localized corrosion or inhibit it depending on surface interactions [31]. Therefore, electrolyte composition must align with the targeted clinical environment and corrosion mechanism (uniform, pitting, galvanic, tribocorrosion, or MIC). Important parameters include pH, ionic composition (chloride, Ca/P, fluoride, sulfide), buffering capacity, organic content, temperature (37 °C), dissolved gases, viscosity, and redox state [32].

To simulate inflammatory conditions, oxidizing agents such as H_2_O_2_ or NaOCl are added to reproduce the effects of ROS, which can significantly increase corrosion rates and alter redox potential, particularly under acidic or hypoxic conditions [33]. Reporting and controlling redox conditions (for instance, by measuring the open-circuit potential (OCP) or E_h_ of the solution), oxygen availability, and aeration status are essential to accurately reproduce oral or bone-interface environments and to assess passive film stability in vitro in a manner consistent with in vivo behavior [34].

## 4. Oral Simulation: Artificial Saliva Solutions

Human saliva is a highly complex and variable biological fluid that cannot be perfectly replicated ex vivo. Therefore, artificial saliva formulations are designed to reproduce its key chemical and physical characteristics in a stable, standardized, and reproducible manner suitable for corrosion studies, because natural saliva rapidly degrades and can support bacterial growth ex vivo. These formulations aim to mimic major inorganic ions and sometimes organic constituents at physiological concentrations and pH. Compositions range from simple electrolyte solutions to more complex mixtures containing multiple ions, organic compounds, and enzymes, with no single universal formula. Most include K^+^, Na^+^, Cl^−^, phosphate, and bicarbonate to simulate buffering capacity, while minor components such as urea or thiocyanate improve chemical realism. Commonly used formulations include Duffó–Quezada, Fusayama–Meyer, Carter–Brugirard, and Ericsson (Table 2) [35].

**Table 2:** Artificial saliva recipes (g/L in deionized water, 37 °C). Values are grams per liter; blank cells indicate the reagent is not used in that formulation. Hydration states are specified in the salt name.

Reagents	Duffó–Quezada Artificial Saliva	Fusayama–Meyer Artificial Saliva	Carter–Brugirard Artificial Saliva	Ericsson’s Artificial Saliva
NaCl	0.600	0.400	0.700	0.584
KCl	0.720	0.400	1.200	-
CaCl_2_·2H_2_O	0.220	0.906	-	-
CaCl_2_	-	-	-	0.166
MgCl_2_	-	-	-	0.014
KH_2_PO_4_	0.680	-	0.260	0.340
Na_2_HPO_4_·12H_2_O	0.856	-	-	-
Na_2_HPO_4_·H_2_O	-	-	0.190	-
Na_2_HPO_4_	-	-	-	0.340
NaH_2_PO_4_·2H_2_O	-	0.690	-	-
NaHCO_3_	-	-	1.500	-
KHCO_3_	1.500	-	-	1.500
KSCN	0.060	-	0.330	-
Urea	-	1.000	0.130	-
Na_2_S·9H_2_O	-	0.005	-	-
Citric acid	0.030	-	-	0.029
pH	~6.5	~5–5.8	~7.6	~6.7

Artificial saliva base formulations are designed to reproduce the inorganic environment governing electrochemical reactions on oral implant surfaces, particularly variables affecting titanium passivity such as chloride activity, buffering capacity, ionic strength, dissolved oxygen availability, and electrical conductivity [35,36]. These parameters directly influence the stability of the TiO_2_ passive film and the kinetics of anodic dissolution and cathodic oxygen reduction. Typical compositions include NaCl and KCl to establish chloride activity and ionic strength, calcium salts to reflect salivary mineral content, phosphate buffers to stabilize pH, and often bicarbonate to reproduce physiological buffering equilibria. Minor constituents such as urea and thiocyanate are incorporated to enhance physiological relevance and simulate metabolic and antimicrobial components of natural saliva. Because electrochemical behavior is highly sensitive to small compositional changes, precise reporting of reagent purity, salt hydration states, preparation sequence, and target pH at 37 °C is essential for interlaboratory reproducibility and meaningful comparison of corrosion data [37].

The inclusion of calcium and phosphate is particularly critical because these ions may precipitate as calcium phosphate phases at the implant surface, altering interfacial chemistry, surface charge, and electrochemical response. Such precipitation can modify impedance spectra, apparent corrosion currents, and passive-film thickness. Calcium-free systems simplify electrochemical interpretation by minimizing mineral deposition, but they underrepresent clinically relevant mineral interactions that occur in vivo. Consequently, electrolyte selection must align with the experimental objective: mineral-inclusive media are appropriate for bioactivity assessment and deposition studies, whereas simpler chloride-buffer systems are preferable when isolating passive-film breakdown, pitting susceptibility, or tribocorrosion mechanisms [38]. Given that TiO_2_ stability is strongly pH-dependent, artificial saliva is typically adjusted to pH 6.0–7.2 to reflect resting oral conditions, with controlled acidification protocols introduced when simulating cariogenic or dietary acid challenges. Parameters such as pH, chloride activity, buffer capacity, and minor additives must be explicitly reported because they significantly influence uniform corrosion, localized attack, and wear-assisted degradation processes [39].

Functionally, artificial saliva systems can be categorized as inorganic-only formulations, organic-adjusted media (e.g., containing urea or thiocyanate), and protein-enriched or rheology-modified systems. Each category is selected according to the dominant mechanism under investigation, including passive-film stability, pellicle formation, microbial biofilm interactions, or tribocorrosion under lubricated conditions [40,41].

Among commonly employed formulations:

• Duffó–Quezada saliva represents a near-neutral composition containing calcium and phosphate together with bicarbonate buffering. This formulation supports mineral equilibria and enables calcium–phosphate deposition at the interface, making it particularly suitable for bioactivity assessment, surface conditioning studies, and investigations of interfacial mineral formation relevant to implant integration [36,42].
• Fusayama–Meyer saliva is slightly acidic and incorporates urea and sulfide species. It is widely used for corrosion and tarnish testing of dental alloys, especially under acidic or fluoride-containing challenge conditions that simulate cariogenic environments or oral hygiene exposures [43,44,45].
• Carter–Brugirard saliva is mildly alkaline (approximately pH 7.6) and deliberately excludes calcium to prevent precipitation and maintain high buffering stability. Its chemical simplicity and reproducibility make it particularly suitable for controlled electrochemical investigations focused on passive-film integrity and breakdown behavior [46].
• Ericsson’s formulation is a simplified inorganic buffer containing calcium and phosphate, commonly used in enamel-focused studies and when isolating ionic and pH effects without interference from organic components [47].

**Figure 2:**
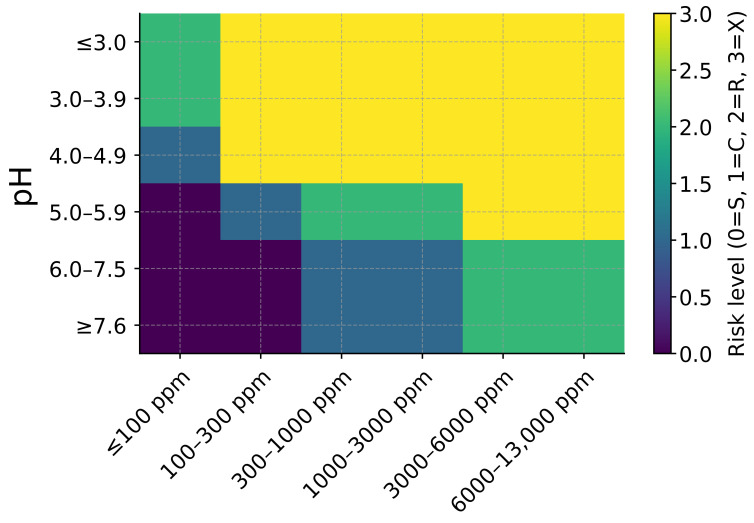
pH–fluoride risk matrix for titanium in fluoride-containing artificial saliva (37 °C). Cells indicate qualitative risk categories: S (safe/passive), C (caution), R (risk of pitting/measurable dissolution), and X (severe/rapid attack). Consistent with the acid–base chemistry of HF (pK_a_ ≈ 3.2), the matrix shows that at pH ≤ 4 even modest fluoride (≈100–300 ppm) can destabilize TiO_2_, whereas around pH 6–7.5 only very high fluoride (≥3000 ppm) is typically problematic. Thresholds are approximate and the risk increases in the presence of chloride, mechanical wear (tribocorrosion), or oxidants (e.g., ROS). (ppm = mg/L·F).

Fluoride-containing artificial saliva is frequently employed to simulate exposure to toothpastes, gels, and professional fluoride treatments. Fluoride concentration and pH jointly determine corrosion behavior, as increasing fluoride levels and decreasing pH act synergistically to destabilize the TiO_2_ passive film, lowering the threshold for passive breakdown and localized attack [48,49]. At high fluoride concentrations (e.g., 5000–10,000 ppm) combined with acidic conditions, corrosion risk increases markedly, whereas neutral fluoride-containing media may promote fluorapatite formation and remineralization processes (Figure 2). Fluoride speciation (F^−^ versus HF) and fluoride source (NaF versus SnF_2_) must be carefully controlled and reported because they exhibit distinct chemical reactivity and electrochemical effects.

Additional additives such as urea, mucin, amylase, and lysozyme are incorporated depending on the targeted mechanism. Urea may influence pH through urease-mediated hydrolysis; mucin modifies viscosity, lubrication, and oxygen transport; amylase can indirectly affect biofilm-driven acid production; and lysozyme alters microbial viability and potentially passive-film behavior. Because these additives can significantly modify corrosion kinetics, tribological response, and electrochemical signatures, their concentration, preparation protocol, storage conditions, and exposure duration must be fully documented to ensure experimental comparability and mechanistic clarity [50,51,52,53].

## 5. Electrolytes Mimicking Physiological Fluids

At the bone–implant interface, corrosion testing relies on physiological electrolytes that simulate interstitial fluid, blood plasma, or tissue environments rather than oral saliva analogs. In contrast to artificial saliva systems, these media emphasize extracellular ionic composition, buffering chemistry, and mineral equilibria characteristic of bone tissue. Commonly employed solutions include Ringer’s solution (representing extracellular saline), phosphate-buffered saline (PBS), Hank’s balanced salt solution (HBSS), Simulated Body Fluid (SBF) and its modified variants, and cell culture media such as DMEM or α-MEM (Table 3). These formulations differ substantially in buffering systems, calcium and phosphate content, organic components, and gas control requirements, all of which influence passive-film stability, ion release, and electrochemical behavior [54,55].

Because electrolyte composition directly governs corrosion thermodynamics and kinetics, variability across suppliers and literature sources must be carefully addressed. Differences in salt hydration states, trace impurities, buffer systems (phosphate, bicarbonate, TRIS), and preparation order can alter ionic strength, pH stability, and supersaturation with respect to calcium phosphates. Therefore, precise reporting of reagent specifications, concentrations (g/L), target pH at 37 °C, and a controlled CO_2_/air atmosphere is essential for reproducibility and accurate interpretation of corrosion results. Failure to document these parameters may lead to significant interlaboratory discrepancies.

Overall, physiological electrolytes span a spectrum from simple saline media to complex organic-containing culture environments, with composition strongly influencing corrosion mechanisms and surface chemistry [56].

Ringer’s solutions are isotonic NaCl-based electrolytes containing K^+^ and Ca^2+^, sometimes buffered with bicarbonate or lactate. They provide relatively mild, chloride-dominated environments suitable for evaluating passive alloys such as titanium, cobalt–chromium, and 316L stainless steel. However, the absence of phosphate and proteins limits physiological fidelity. Bicarbonate-buffered variants require controlled CO_2_ atmospheres to maintain stable pH and may promote CaCO_3_ precipitation, whereas lactated Ringer’s is more stable under ambient conditions. For passive alloys, Ringer’s solution is generally non-aggressive, yet it may overestimate corrosion by lacking minor ionic species and organic components present in vivo [57,58].

Phosphate-buffered saline (PBS) is widely adopted because it maintains stable physiological pH (~7.2–7.4) without the need for CO_2_ equilibration. Phosphate buffering provides reproducible conditions for assessing passive-film integrity, pitting susceptibility, and uniform dissolution kinetics. Many PBS formulations exclude Ca^2+^ and Mg^2+^ to prevent precipitation, thereby simplifying electrochemical analysis. While PBS effectively captures thermodynamic drivers such as chloride activity and pH, it lacks proteins and complexing agents that influence corrosion kinetics, and thus may slightly overestimate metal release compared with actual plasma conditions [59].

Hank’s balanced salt solution (HBSS) more closely replicates extracellular ionic composition by incorporating bicarbonate, phosphate, Ca^2+^, and Mg^2+^. This improved chemical fidelity enhances relevance for endosseous implant evaluation. However, bicarbonate-containing HBSS requires CO_2_ control to maintain physiological pH unless alternative buffers such as HEPES are introduced. The presence of divalent cations permits calcium phosphate or carbonate precipitation, which can modify surface charge, alter electrochemical impedance, and complicate interpretation of corrosion currents. Because multiple HBSS variants exist, detailed reporting of the formulation is critical [60].

Simulated body fluids (SBFs) are formulated to match human plasma ionic ratios and are intentionally slightly supersaturated with respect to hydroxyapatite. Classic Kokubo SBF uses TRIS buffering at pH ~7.25, while modified SBF versions adjust bicarbonate concentration or ion content to better replicate plasma chemistry. SBF promotes calcium phosphate deposition on titanium and other implant alloys, which is advantageous for evaluating bioactivity and osseointegration potential. However, this mineral deposition couples electrochemical oxidation with precipitation phenomena, complicating quantitative corrosion analysis. Despite these challenges, SBF remains highly relevant for endosseous implant testing due to its close approximation of plasma mineral equilibria [61,62].

Cell culture media (DMEM, α-MEM, MEM) contain inorganic salts plus amino acids, vitamins, glucose, and often serum proteins. These media provide greater biochemical realism, influencing corrosion via adsorption, chelation, redox reactions, and protein film formation. Proteins may inhibit or accelerate corrosion depending on conditions, and sterility is critical to prevent microbial-induced artifacts. Corrosion rates of passive alloys are generally low but mechanistically distinct from purely inorganic media [63,64].

**Table 3:** Compositions of physiological simulants used in corrosion testing (g/L in 1 L deionized water at 37 °C). Hydration states are shown in the reagent name; blank cells indicate the component is not present in that formulation. For SBF/m-SBF, TRIS mass and the volume of 1 M HCl refer to adjustment to pH 7.40 at 36–37 °C. DMEM and α-MEM values correspond to typical 1× powders; pH is maintained under 5% CO_2_ unless otherwise stated.

Reagents	Ringer’s	PBS 1× (No Ca/Mg)	PBS 1× (+Ca/Mg)	HBSS 1× (with Ca/Mg)	SBF (Kokubo 1×, TRIS)	r-SBF (27 mM HCO_3_^−^)	DMEM 1× (High Glucose)	α-MEM 1×	SBP
NaCl	8.6	8.0	8.0	8.0	8.035	6.547	6.4	6.8	6.8
KCl	0.3	0.2	0.2	0.4	0.225	0.373	0.4	0.4	0.4
NaHCO_3_	-	-	-	0.35	0.355	2.268	3.7	2.2	2.2
Na_2_SO_4_	-	-	-	-	0.072	0.071	-	-	-
Na_2_HPO_4_ (anhydrous)	-	1.44	1.44	0.048	-	-	-	-	0.126
Na_2_HPO_4_·2H_2_O	-	-	-	-	-	0.178	-	-	-
NaH_2_PO_4_ (anhydrous)	-	-	-	-	-	-	-	-	0.026
NaH_2_PO_4_·H_2_O	-	-	-	-	-	-	0.125	0.14	-
KH_2_PO_4_	-	0.24	0.24	0.06	-	-	-	-	-
K_2_HPO_4_·3H_2_O	-	-	-	-	0.231	-	-	-	-
CaCl_2_ (anhydrous)	-	-	0.132	-	0.292	-	0.2	-	0.2
CaCl_2_·2H_2_O	0.33	-	-	0.185	-	0.368	-	0.265	-
MgCl_2_ (anhydrous)	-	-	-	0.1	-	-	-	-	-
MgCl_2_·6H_2_O	-	-	0.102	-	0.311	0.305	-	-	-
MgSO_4_ (anhydrous)	-	-	-	0.098	-	-	0.098	0.098	0.1
D-Glucose	-	-	-	1.0	-	-	4.5	1.0	-
TRIS	-	-	-	-	6.118	6.057	-	-	-
HEPES	-	-	-	2.38	-	-	-	-	-
Phenol red	-	-	-	-	-	-	0.015	-	-
pH	7.3–7.4	7.3–7.4	7.3–7.4	~7.4	7.4	7.4	~7.4	~7.4	7.36
pH adjustment	-	Titrate with HCl/NaOH to pH 7.4 at 37 °C	Titrate with HCl/NaOH to pH 7.4 at 37 °C	Bicarbonate/CO_2_ buffering; equilibrate under 5% CO_2_	TRIS/HCl system; add ~39 mL/L of 1 M HCl at 36.5–37 °C to pH 7.40	TRIS/HCl system; add ~15 mL/L of 1 M HCl at 36.5–37 °C to pH 7.40	Bicarbonate/CO_2_ buffering; equilibrate under 10% CO_2_	Bicarbonate/CO_2_ buffering	Bicarbonate/CO_2_ buffering; equilibrate as needed
CO_2_ requirement	-	-	-	5% CO_2_	-	Optional (closed system or 5% CO_2_ to stabilize HCO_3_^−^)	10% CO_2_	5–10% CO_2_	5% CO_2_

Synthetic Blood Plasma (SBP) is a high-fidelity inorganic plasma analog containing full physiological bicarbonate (~27 mM) and requiring CO_2_ equilibration at 37 °C. It closely reproduces in vivo ionic conditions and is often specified in standards. SBP better correlates with clinical corrosion behavior than simple saline, though it lacks proteins unless specifically supplemented [65].

Overall, corrosion behavior depends strongly on electrolyte complexity. Simpler solutions (Ringer’s solution and PBS) offer reproducibility and mechanistic clarity, whereas SBF, SBP, and cell culture media provide greater physiological realism but introduce mineral deposition, buffering dynamics, and organic interactions that complicate interpretation. Accurate reporting of composition, buffering system, CO_2_ control, and temperature (37 °C) is essential for meaningful comparison across studies.

## 6. Challenge Media: Acidity, Alkalinity, Oxidants, and Additives

Beyond simulating normal physiological fluids, corrosion studies often use challenge media that replicate extreme or pathological conditions such as infection, inflammation, dietary acid exposure, or medical treatments. These environments test the stability of an implant’s passive film under adverse chemistry. Common categories include acidic media (dietary or clinical acids), metabolic alterations linked to disease or obesity, alkaline conditions caused by urease activity and ammonia production, and oxidative environments containing peroxides or reactive oxygen species. Evaluating corrosion under these stress conditions is critical for assessing material durability under diverse patient-relevant scenarios across diverse patient scenarios, not only under ideal homeostatic conditions [39].

### 6.1. Dietary and Clinical Acids

Implant materials in the oral and peri-implant environment are frequently exposed to acidic conditions arising from both dietary intake and clinical interventions. Common dietary acids include citric acid from citrus fruits, phosphoric acid in carbonated beverages, and acetic acid in vinegar. These acids can temporarily lower local pH around dental implants, prosthetic components, or orthodontic wires. In clinical practice, acids are also intentionally applied. Citric acid solutions (typically 20–40%) are used to decontaminate titanium surfaces during peri-implantitis therapy, while phosphoric acid gels (~30–40%) are employed for enamel etching and may contact metallic restorations. Such exposures challenge the passive oxide films of metals either by direct acid dissolution or by complexing released metal ions, thereby altering dissolution equilibria [66].

Citric acid is particularly notable because it combines acidity with strong chelating ability. As a tricarboxylic acid, it forms stable complexes with transition-metal ions. Under near-neutral conditions, citrate anions can adsorb to metallic surfaces and even inhibit corrosion [67]. Studies in artificial saliva have shown that moderate citric acid additions (up to ~9 g/L) can improve corrosion resistance of 316L stainless steel by forming protective iron- or chromium-citrate surface complexes. This inhibition mechanism relies on adsorption and surface blocking at buffered pH. However, at low pH (<3), citric acid becomes predominantly protonated (H_3_Cit) and acts aggressively, dissolving passive oxides. Clinically applied citric acid gels remove biofilm and partially dissolve titanium oxide, leaving a microscopically roughened surface that rapidly repassivates after rinsing. In fact, citric acid is widely used in industry for stainless steel passivation because it removes free iron and promotes chromium oxide formation. Short exposures may temporarily increase metal ion release but often leave a clean and corrosion-resistant surface after repassivation [68].

Phosphoric acid represents a stronger acidic challenge. Present in soft drinks (pH ≈ 2.5) and concentrated dental etchants (≈37% H_3_PO_4_), it primarily attacks passive films through low pH rather than strong chelation. In stainless steel and cobalt–chromium alloys, concentrated phosphoric acid can dissolve Cr_2_O_3_ films during prolonged contact. In the oral cavity, transient exposures to acidic beverages cause temporary depassivation until saliva restores neutrality. Although phosphate species may form protective metal phosphates under certain conditions, at very low pH metal oxides become soluble. Controlled phosphoric acid cleaning can be effective, but direct contact with concentrated gels should be minimized and followed by thorough rinsing [69,70].

Acetic acid, the active component of vinegar (3–5%, pH ≈ 2.4), is a weaker, non-oxidizing acid. It tends to cause uniform dissolution rather than pitting. Stainless steels exposed to mildly acidic acetate solutions may dissolve until oxide regeneration occurs. Acetate ions can weakly adsorb and sometimes inhibit corrosion, though less effectively than citrate. Compared with hypochlorite-based disinfectants, acetic acid typically produces less corrosion on CoCr alloys. Moderate exposure generally results in minor passive-film thinning rather than severe localized attack [71].

Overall, corrosion rates increase markedly as pH decreases. Titanium’s TiO_2_ film dissolves more rapidly below pH 4, and Ti–6Al–4V exhibits substantially higher corrosion rates at extreme acidity [72]. Stainless steels become actively corroding below pH 2 in chloride-containing media, and CoCr alloys also lose passivity under such conditions [73]. Testing in acidic challenge media is therefore essential for evaluating resistance to transient acid exposure. Although Ti, CoCr, and 316L stainless steel are generally resistant to short-term acid exposure, repeated acid attacks may cause episodic metal ion release relevant to biological responses.

### 6.2. Metabolic Disorders and Drug Effects

Systemic diseases and medications can significantly alter the chemical microenvironment around implants. Obesity, diabetes, and metabolic syndrome are associated with chronic inflammation, elevated glucose, increased free fatty acids (FFAs), and oxidative stress. Renal or hepatic dysfunction can introduce atypical metabolites such as excess urea, ammonium, or altered amino acid profiles. These changes influence pH, oxidant levels, and metal-ion complexation, potentially destabilizing passive films [74,75,76].

Free fatty acids such as palmitic, oleic, and stearic acid are elevated in obesity. While not strongly corrosive themselves, FFAs can adsorb onto implant surfaces or incorporate into biofilms, altering wettability and oxygen transport [77]. Adsorbed lipid layers may create occluded regions that trap corrosive agents or hinder repassivation. Oxidized lipids, including lipid peroxides formed during oxidative stress, may act as mild oxidants capable of initiating localized oxide attack. Fatty acids can also bind released metal ions, forming organometallic soaps (e.g., cobalt stearate), influencing metal transport and possibly corrosion kinetics. Although their direct corrosive effect is modest, their combined presence with oxidative stress may contribute to implant degradation [78,79].

Pharmaceuticals and metabolites further complicate corrosion behavior. Sulfur-containing compounds can elevate sulfide levels, which are known to destabilize passive films on stainless steels and CoCr alloys. Inflammatory drugs or disease states that increase peroxide production enhance oxidative stress. Ketoacids in poorly controlled diabetes lower pH, while advanced glycation end-products (AGEs) sustain inflammation. These combined chemical changes may explain observations of elevated systemic metal-ion levels in certain patient populations [80].

### 6.3. Alkaline Challenges: Urease and Ammonia

Alkaline environments present distinct corrosion mechanisms. Urease-producing bacteria (e.g., Proteus, Klebsiella) hydrolyze urea into ammonia and carbonate, raising local pH to 8–9 [81]. Such alkalization can occur near infected implants. Titanium alloys remain highly stable in alkaline conditions, with TiO_2_ maintaining passivity up to pH 12–13. Moderate alkalinity often enhances repassivation and reduces hydrogen evolution. However, ammonia can form soluble ammine complexes with Ni^2+^ and Co^2+^, sustaining dissolution in stainless steels and CoCr alloys by preventing hydroxide precipitation. Thus, ammonia-rich environments may increase metal-ion release even when overall corrosion potentials shift in the noble direction. Urease-based in vitro models have demonstrated pH increases and increased nickel release in such systems.

### 6.4. Peroxides and Reactive Oxygen Species (ROS)

Oxidative stress during inflammation is among the most aggressive in vivo challenges. Activated immune cells release hydrogen peroxide (H_2_O_2_), hypochlorous acid (HOCl), superoxide, and hydroxyl radicals. H_2_O_2_ at millimolar levels can alter titanium oxide chemistry, forming peroxo or hydrated species that increase oxide conductivity and dissolution. Experimental addition of H_2_O_2_ significantly elevates passive currents and reduces impedance of Ti alloys. HOCl is even more aggressive, capable of raising corrosion potentials and increasing current densities by orders of magnitude in CoCr alloys. It promotes transpassive oxidation and severe surface etching [82,83,84]. Fenton-type reactions involving Fe^2+^ and H_2_O_2_ generate hydroxyl radicals, further accelerating corrosion. Because ROS occur in bursts in vivo, laboratory protocols often simulate transient oxidative pulses rather than continuous exposure [85].

### 6.5. Serum Proteins

Proteins such as albumin, globulins, fibrinogen, and fibronectin rapidly adsorb to implant surfaces. Through the Vroman effect, adsorption is dynamic and competitive. Protein layers influence corrosion by acting as physical barriers to aggressive ions, binding dissolved metal ions (maintaining dissolution driving force), and altering oxygen transport. Adsorbed proteins may inhibit uniform corrosion but can also create differential aeration cells, promoting localized attack. In tribocorrosion, proteins can lubricate surfaces while simultaneously delaying oxide regrowth [86,87].

### 6.6. Organic Complexing Agents

Small anions such as citrate, lactate, and phosphate influence corrosion through complexation and surface interactions. Citrate is a strong chelator that generally promotes dissolution by stabilizing metal ions in solution. Lactate is weaker but may modestly enhance ion solubility. Phosphate, in contrast, typically acts as a corrosion inhibitor by adsorbing to oxide surfaces, suppressing chloride attack, and forming protective precipitates. Consequently, phosphate-buffered media are often less aggressive than simple saline solutions [88,89].

## 7. Standards, Comparability, and Reproducibility

Corrosion and ion-release testing of dental alloys such as commercially pure titanium, Ti–6Al–4V, cobalt–chromium, and stainless steels is guided by established international standards. ISO 10271 provides the principal dental-specific framework for immersion and electrochemical corrosion testing in standardized electrolytes, while ISO 10993-15 focuses on metal ion release, extract preparation, analytical quantification, and reporting for medical and dental devices.

Several ASTM standards complement these guidelines. ASTM G31 addresses immersion testing for mass loss and surface evaluation; ASTM G5 and ASTM G59 describe potentiodynamic polarization methods for determining corrosion current density and passivation behavior; ASTM G61 and ASTM F2129 define cyclic polarization techniques for assessing susceptibility to localized corrosion and passive film breakdown, particularly relevant under fluoride, acidic, or chloride-rich conditions; and ASTM F746 addresses pitting and crevice corrosion of implant materials. Although these standards provide robust methodologies, their default electrolytes and protocols often require adaptation to simulate clinically relevant oral or endosseous chemistries, especially when fluoride, proteins, carbonate buffering, or biofilms are involved.

To ensure reproducibility and cross-laboratory comparability, studies should comprehensively report electrolyte composition (including reagent source, hydration state, concentration, and preparation sequence), and target and measure pH at 37 °C, redox potential (Eh), dissolved oxygen, and gas atmosphere (e.g., CO_2_ control for bicarbonate-buffered systems). When organics or proteins are included, their source, purity, and concentration must be specified. Hydrodynamic conditions (flow, shear rate, agitation, temperature tolerance), sterilization procedures, and microbial controls (for MIC studies) should also be detailed.

Electrochemical protocols must clearly state electrode configuration, reference electrode calibration, scan parameters, resistance compensation, and stabilization criteria. Immersion tests should report exposed surface area, finish, preconditioning history, and quantitative mass-loss methods with detection limits. Transparent documentation of fluoride levels, pH conditions, and MIC-related parameters (e.g., sulfide concentration, oxygen profiles) is essential for meaningful comparison and meta-analysis of corrosion behavior in dental implant materials under simulated oral and bone environments.

## 8. Limitations of Simulated Fluids and Clinical Translation

Although simulated fluids are essential for controlled corrosion testing of dental implant materials, several limitations must be acknowledged when interpreting in vitro findings. A major limitation is the oversimplification of chemical composition. Many commonly used electrolytes reproduce only selected inorganic ions, pH, chloride concentration, or buffering systems, while omitting or simplifying salivary proteins, enzymes, organic acids, sulfur-containing species, lipids, metabolites, and inflammatory mediators. Therefore, simplified solutions such as NaCl, PBS, Ringer’s solution, or basic artificial saliva provide useful mechanistic information but cannot fully reproduce the biochemical complexity of the oral cavity or peri-implant tissues.

Another important limitation is the incomplete simulation of the oral microbiota. Natural peri-implant biofilms are multispecies, spatially organized, and metabolically heterogeneous. They generate local gradients in pH, oxygen concentration, redox potential, sulfide concentration, and reactive oxygen species. Most in vitro corrosion studies either omit microorganisms entirely or use simplified monoculture or short-term biofilm models. These approaches are useful for isolating specific mechanisms but do not fully reproduce mature peri-implant biofilms or the complex interactions among microbial metabolism, inflammation, passive-film degradation, and metal-ion release.

Mechanical, hydrodynamic, and thermal factors are also incompletely represented in many electrolyte-based corrosion studies. In vivo, dental implants are exposed to mastication, micromotion at the implant–abutment interface, fretting, wear, salivary flow, cyclic wetting and drying, and temperature fluctuations caused by food and beverages. These factors may disrupt passive films, alter oxygen transport, influence protein adsorption, and accelerate tribocorrosion. Static immersion or conventional electrochemical tests may therefore underestimate or misrepresent corrosion processes occurring under clinically relevant mechanical and thermal cycling.

A further limitation is the lack of a validated quantitative framework linking in vitro corrosion parameters to clinical outcomes. Electrochemical metrics such as open-circuit potential, corrosion current density, breakdown potential, impedance response, and ion-release concentration provide valuable mechanistic information, but they cannot yet be directly translated into predictable clinical endpoints such as peri-implantitis incidence, peri-implant marginal bone loss, soft-tissue inflammation, implant mobility, or implant failure. Peri-implant disease is multifactorial, involving biofilm accumulation, host immune response, implant surface topography, prosthetic design, mechanical loading, systemic health, surgical variables, and patient-specific oral hygiene.

The present article should also be interpreted in light of its review design. It is a structured narrative review rather than a systematic review or meta-analysis. Therefore, no formal risk-of-bias assessment, evidence-quality grading, or quantitative pooling of corrosion outcomes was performed. The available evidence is also highly heterogeneous, with major differences in electrolyte composition, pH, fluoride concentration, chloride content, protein supplementation, oxygen control, redox conditions, temperature, hydrodynamics, surface preparation, alloy composition, exposure duration, and electrochemical protocols. This heterogeneity limits direct comparison among studies and prevents the definition of universal corrosion thresholds for clinical risk assessment.

Consequently, the findings summarized in this review should be interpreted primarily as mechanistic guidance for electrolyte selection and experimental design, rather than as direct predictors of long-term clinical implant performance. Future research should integrate standardized in vitro corrosion protocols with dynamic pH cycling, thermal cycling, controlled flow, protein supplementation, clinically relevant biofilm models, tribocorrosion testing, and longitudinal clinical endpoints. Combining corrosion metrics with peri-implant tissue metal content, inflammatory biomarkers, microbiome composition, radiographic bone-level changes, and implant survival data would improve the translational value of this research field.

## 9. Conclusions and Future Directions

Electrolyte selection fundamentally shapes the interpretation of corrosion behavior for titanium, cobalt–chromium, and stainless steel dental implants under simulated oral and endosseous conditions. Artificial saliva formulations are most appropriate for supragingival and peri-gingival simulations, particularly when evaluating acidity, fluoride exposure, organic additives, pitting susceptibility, and passive-film stability. In contrast, buffered physiological solutions such as PBS, HBSS, and synthetic plasma are more suitable for endosseous simulations focused on ion release and passive durability in extracellular-fluid-like environments. When calcium–phosphate deposition or bioactivity is the target, SBF variants are useful, whereas cell culture media such as DMEM or α-MEM are better suited for studies involving proteins, organic complexation, and long-term surface fouling. For microbiologically influenced corrosion, enriched saliva or saliva–broth systems under controlled oxygen and redox conditions provide more biologically relevant test environments.

Across all test types, reproducibility requires detailed reporting of electrolyte composition, reagent identity and hydration state, preparation sequence, pH at 37 °C, gas equilibration, dissolved oxygen, redox potential, hydrodynamic conditions, sterilization procedures, electrochemical configuration, exposed area, and surface finish. A shared reporting checklist would improve cross-laboratory comparison and support future meta-analyses.

Future progress should focus on dynamic and clinically relevant test platforms that reproduce the temporal variability of the oral environment. Programmable systems capable of cycling pH, fluoride concentration, reactive oxygen species, temperature, flow, and oxygen availability could better simulate meal-induced acidification, fluoride exposure from oral hygiene products, and inflammatory oxidative bursts. Coupled biofilm–tribocorrosion models are also needed to clarify interactions among microbial metabolism, extracellular polymeric substances, micromotion, passive-film disruption, and metal-ion release.

Finally, harmonized datasets combining electrolyte metadata, polarization curves, impedance spectra, ion-release profiles, surface characterization, and biological responses could support predictive modeling and improve the translational value of corrosion studies. However, until quantitative links between in vitro corrosion metrics and clinical outcomes are established, simulated-fluid studies should be interpreted primarily as mechanistic tools rather than direct predictors of peri-implantitis, peri-implant bone loss, or implant failure. Integrating standardized corrosion testing with clinically relevant biological and longitudinal data remains a key priority for future research.

## Figures and Tables

**Table 1:** Major inorganic ions in human saliva versus blood plasma (mM, 37 °C). Salivary values represent typical ranges reported for unstimulated and stimulated whole saliva. Concentrations in plasma are physiological averages. Salivary bicarbonate and chloride vary strongly with flow rate. Ranges were compiled from reviews; salivary values vary with flow rate and gland source.

Ion	Human Saliva (mM)	Blood Plasma (mM)	Notes
Na^+^	Unstimulated: 2–21; Stimulated: 10–80	136–146	Saliva rises with flow rate
K^+^	10–30	3.5–5.0	Higher in saliva than plasma
Ca^2+^ (free)	1.0–2.5	1.1–1.3 (total plasma Ca ≈ 2.2–2.6 mM)	Comparable free Ca^2+^ levels
Mg^2+^	0.2–0.6	0.7–1.1	-
Cl^−^	5–40	95–105	Saliva increases with flow rate
HCO_3_^−^	1–60	22–28	Strongly flow-dependent in saliva
PO_4_^3−^ (inorganic)	2–10	0.8–1.5	Mostly as HPO_4_^2−^/H_2_PO_4_^−^
SCN^−^	0.5–3 (non-smokers); up to 6 (smokers)	-	Highly diet/smoking dependent
F^−^	0.02–0.05	-	Depends on fluoridation; ≈0.4–1 ppm

## Data Availability

No new data were created or analyzed in this study.

## References

[1] Pandey C., Rokaya D., Bhattarai B.P. (2022). Contemporary concepts in osseointegration of dental implants: A review. Biomed. Res. Int..

[2] Rushinek H., Cohen A., Casap N., Alterman M. (2025). The effect of implant-associated factors on the long-term outcomes of dental implants. Oral. Maxillofac. Surg. Clin..

[3] Kreth J., Merritt J., Pfeifer C.S., Khajotia S., Ferracane J.L. (2020). Interaction between the oral microbiome and dental composite biomaterials: Where we are and where we should go. J. Dent. Res..

[4] Kim J.C., Lee M., Yeo I.S.L. (2022). Three interfaces of the dental implant system and their clinical effects on hard and soft tissues. Mater. Horiz..

[5] Chmielewski M., Pilloni A. (2023). Current molecular, cellular and genetic aspects of peri-implantitis disease: A narrative review. Dent. J..

[6] Nagay B.E., Cordeiro J.M., Barao V.A. (2022). Insight into corrosion of dental implants: From biochemical mechanisms to designing corrosion-resistant materials. Curr. Oral. Health Rep..

[7] Lechner J., Noumbissi S., von Baehr V. (2018). Titanium implants and silent inflammation in jawbone—A critical interplay of dissolved titanium particles and cytokines TNF-α and RANTES/CCL5 on overall health?. EPMA J..

[8] Bressan E., Ferroni L., Gardin C., Bellin G., Sbricoli L., Sivolella S., Zavan B. (2019). Metal nanoparticles released from dental implant surfaces: Potential contribution to chronic inflammation and peri-implant bone loss. Materials.

[9] Bordbar-Khiabani A., Gasik M. (2023). Electrochemical behavior of additively manufactured patterned titanium alloys under simulated normal, inflammatory, and severe inflammatory conditions. J. Mater. Res. Technol..

[10] Bordbar-Khiabani A., Gasik M. (2023). Electrochemical and biological characterization of Ti–Nb–Zr–Si alloy for orthopedic applications. Sci. Rep..

[11] Bordbar-Khiabani A., Bahrampour S., Mozafari M., Gasik M. (2022). Surface functionalization of anodized tantalum with Mn_3_O_4_ nanoparticles for effective corrosion protection in simulated inflammatory condition. Ceram. Int..

[12] Kim S.M. (2023). Oral galvanism related to dental implants. Maxillofac. Plast. Reconstr. Surg..

[13] Thiyaneswaran N., Rahul B., Devi S., Devi M.S., Selvaganesh S. (2023). Micro gap at implant abutment connections—A systematic review. J. Pharm. Sci. Res..

[14] Kheder W., Al Kawas S., Khalaf K., Samsudin A.R. (2021). Impact of tribocorrosion and titanium particles release on dental implant complications—A narrative review. Jpn. Dent. Sci. Rev..

[15] Vegas-Bustamante E., Sanmartí-García G., Gil J., Delgado-Garoña L., Figueiredo R., Camps-Font O., Toledano-Serrabona J. (2025). Effect of tribocorrosion on mechanical behavior of titanium dental implants: An in vitro study. Materials.

[16] Hubenova E., Mitov M., Hubenova Y. (2024). Microbiologically influenced corrosion can cause a dental implant rejection. Electrochim. Acta.

[17] Costa R.C., Abdo V.L., Mendes P.H., Mota-Veloso I., Bertolini M., Mathew M.T., Souza J.G.S. (2021). Microbial corrosion in titanium-based dental implants: How tiny bacteria can create a big problem?. J. Bio-Tribo-Corros..

[18] De Stefano M., Aliberti S.M., Ruggiero A. (2022). (Bio) tribocorrosion in dental implants: Principles and techniques of investigation. Appl. Sci..

[19] Bordbar-Khiabani A., Ebrahimi S., Yarmand B. (2020). In-vitro corrosion and bioactivity behavior of tailored calcium phosphate-containing zinc oxide coating prepared by plasma electrolytic oxidation. Corros. Sci..

[20] Bordbar-Khiabani A., Yarmand B., Sharifi-Asl S., Mozafari M. (2020). Improved corrosion performance of biodegradable magnesium in simulated inflammatory condition via drug-loaded plasma electrolytic oxidation coatings. Mater. Chem. Phys..

[21] Eliaz N. (2019). Corrosion of metallic biomaterials: A review. Materials.

[22] Alhamad M., Barao V.A.R., Sukotjo C., Yerokhin A., Mathew M.T. (2023). Unpredictable electrochemical processes in Ti dental implants: The role of Ti ions and inflammatory products. ACS Appl. Bio Mater..

[23] Jamali R., Bordbar-Khiabani A., Yarmand B., Mozafari M., Kolahi A. (2022). Effects of co-incorporated ternary elements on biocorrosion stability, antibacterial efficacy, and cytotoxicity of plasma electrolytic oxidized titanium for implant dentistry. Mater. Chem. Phys..

[24] Mohammed N.B., Daily Z.A., Alsharbaty M.H., Abullais S.S., Arora S., Lafta H.A., Jalil A.T., Almulla A.F., Ramírez-Coronel A.A., Aravindhan S. (2022). Effect of PMMA sealing treatment on the corrosion behavior of plasma electrolytic oxidized titanium dental implants in fluoride-containing saliva solution. Mater. Res. Express.

[25] Chifor E., Bordeianu I., Anastasescu C., Calderon-Moreno J.M., Bratan V., Eftemie D.-I., Anastasescu M., Preda S., Plavan G., Pelinescu D. (2022). Bioactive coatings based on nanostructured TiO_2_ modified with noble metal nanoparticles and lysozyme for Ti dental implants. Nanomaterials.

[26] Larsen M.J., Pearce E.I.F. (2003). Saturation of human saliva with respect to calcium salts. Arch. Oral. Biol..

[27] Pytko-Polonczyk J., Jakubik A., Przeklasa-Bierowiec A., Muszynska B. (2017). Artificial saliva and its use in biological experiments. J. Physiol. Pharmacol..

[28] Baumann T., Kozik J., Lussi A., Carvalho T.S. (2016). Erosion protection conferred by whole human saliva, dialysed saliva, and artificial saliva. Sci. Rep..

[29] Yilmaz B., Pazarceviren A.E., Tezcaner A., Evis Z. (2020). Historical development of simulated body fluids used in biomedical applications: A review. Microchem. J..

[30] Marco I., Feyerabend F., Willumeit-Römer R., Van der Biest O. (2016). Degradation testing of Mg alloys in Dulbecco’s modified Eagle medium: Influence of medium sterilization. Mater. Sci. Eng. C.

[31] Wagener V., Virtanen S. (2017). Influence of electrolyte composition (simulated body fluid vs. Dulbecco’s modified Eagle’s medium), temperature, and solution flow on the biocorrosion behavior of commercially pure Mg. Corrosion.

[32] Kokubo T., Yamaguchi S. (2019). Simulated body fluid and the novel bioactive materials derived from it. J. Biomed. Mater. Res. Part A.

[33] Prestat M., Thierry D. (2021). Corrosion of titanium under simulated inflammation conditions: Clinical context and in vitro investigations. Acta Biomater..

[34] Gilbert J.L., Kubacki G.W. (2016). Oxidative stress, inflammation, and the corrosion of metallic biomaterials: Corrosion causes biology and biology causes corrosion. Oxidative Stress and Biomaterials.

[35] Preetha A., Banerjee R. (2005). Comparison of artificial saliva substitutes. Trends Biomater. Artif. Organs.

[36] Duffo G.S., Castillo E.Q. (2004). Development of an artificial saliva solution for studying the corrosion behavior of dental alloys. Corrosion.

[37] Buruiana D.L., Bogatu N.L., Muresan A.C., Herbei E.E., Trus C., Ghisman V. (2025). Evaluating the impact of artificial saliva formulations on stainless steel integrity. Appl. Sci..

[38] De Aguiar S.R.M.M., Nicolai M., Almeida M., Gomes A. (2015). Electrochemical behaviour of a cobalt–chromium–molybdenum dental alloy in artificial salivas: Influence of phosphate ions and mucin components. Biomed. Mater. Eng..

[39] Souza J.C., Apaza-Bedoya K., Benfatti C.A., Silva F.S., Henriques B. (2020). A comprehensive review on the corrosion pathways of titanium dental implants and their biological adverse effects. Metals.

[40] Biggio D., Fantauzzi M., Elsener B., Atzei D., Rossi A. (2023). The role of organic compounds in artificial saliva for corrosion studies: Evidence from X-ray photoelectron spectroscopy. Surf. Interface Anal..

[41] Biggio D., Elsener B., Usai G., Fantauzzi M., Rossi A. (2024). Surface chemistry of passive films on Ni-free stainless steel: The effect of organic components in artificial saliva. Langmuir.

[42] Solá C., Amorim A., Espías Á., Capelo S., Fernandes J., Proenga L., Sanchez L., Fonseca I. (2013). Galvanic corrosion behaviour of Ti and Ti6Al4V coupled to noble dental alloys. Int. J. Electrochem. Sci..

[43] Fusayama T., Katayori T., Nomoto S. (1963). Corrosion of gold and amalgam placed in contact with each other. J. Dent. Res..

[44] Meyer J.M., Nally J.N. (1975). Influence of artificial salivas on corrosion of dental alloys. J. Dent. Res..

[45] Gal J.Y., Fovet Y., Adib-Yadzi M. (2001). About a synthetic saliva for in vitro studies. Talanta.

[46] Popa M.V., Vasilescu E., Drob P., Vasilescu C., Demetrescu I., Ionita D. (2008). Long-term assessment of the implant titanium material—Artificial saliva interface. J. Mater. Sci. Mater. Med..

[47] Ericsson Y. (1959). Clinical investigations of the salivary buffering action. Acta Odontol. Scand..

[48] Slama H., Tayyaba Q., Kadiri M., Hermawan H. (2025). Electrochemical evaluation of new Ti-based high-entropy alloys in artificial saliva with fluoride: Implications for dental implant applications. Materials.

[49] Kakaa F., Ferkhi M., Khaled A., Amira S., Eyraud M. (2025). Corrosion behavior of electrochemical and thermal treated titanium into artificial saliva: Effect of pH and fluoride concentration. Corros. Mater. Degrad..

[50] Bilhan H., Bilgin T.A.F.C., Cakir A.F., Yuksel B., Von Fraunhofer J.A. (2007). The effect of mucine, IgA, urea, and lysozyme on the corrosion behavior of various non-precious dental alloys and pure titanium in artificial saliva. J. Biomater. Appl..

[51] Smart P., Neville A., Bryant M. (2020). Tribocorrosion of dental tissues: The role of mucin. Tribol. Int..

[52] Mystkowska J., Łysik D., Klekotka M. (2019). Effect of saliva and mucin-based saliva substitutes on fretting processes of 316 austenitic stainless steel. Metals.

[53] Zhang C., Liu J., Yu W., Sun D., Sun X. (2015). Susceptibility to corrosion of laser welding composite arch wire in artificial saliva of salivary amylase and pancreatic amylase. Mater. Sci. Eng. C.

[54] Insua A., Monje A., Wang H.L., Miron R.J. (2017). Basis of bone metabolism around dental implants during osseointegration and peri-implant bone loss. J. Biomed. Mater. Res. Part A.

[55] Bordbar-Khiabani A., Kovrlija I., Locs J., Loca D., Gasik M. (2023). Octacalcium phosphate-laden hydrogels on 3D-printed titanium biomaterials improve corrosion resistance in simulated biological media. Int. J. Mol. Sci..

[56] Ghodrati H., Goodarzi A., Golrokhian M., Fattahi F., Anzabi R.M., Mohammadikhah M., Mirhadi S. (2025). A narrative review of recent developments in osseointegration and anti-corrosion of titanium dental implants with nano surface. Bone Rep..

[57] Almeraya-Calderón F., Jáquez-Muñoz J.M., Lara-Banda M., Zambrano-Robledo P., Cabral-Miramontes J.A., Lira-Martínez A., Tiburcio C.G. (2022). Corrosion behavior of titanium and titanium alloys in Ringer’s solution. Int. J. Electrochem. Sci..

[58] Kumar S., Narayanan T.S. (2009). Electrochemical characterization of β-Ti alloy in Ringer’s solution for implant application. J. Alloys Compd..

[59] Gudić S., Vrsalović L., Kvrgić D., Nagode A. (2021). Electrochemical behaviour of Ti and Ti-6Al-4V alloy in phosphate buffered saline solution. Materials.

[60] Msweli N.P., Akinwamide S.O., Olubambi P.A., Obadele B.A. (2023). Microstructure and biocorrosion studies of spark plasma sintered yttria stabilized zirconia reinforced Ti6Al7Nb alloy in Hanks’ solution. Mater. Chem. Phys..

[61] Baino F., Yamaguchi S. (2020). The use of simulated body fluid (SBF) for assessing materials bioactivity in the context of tissue engineering: Review and challenges. Biomimetics.

[62] Groeger S., Meyle J. (2021). Reactivity of titanium dental implant surfaces in simulated body fluid. ACS Appl. Bio Mater..

[63] Tas A.C. (2014). Grade-1 titanium soaked in a DMEM solution at 37 °C. Mater. Sci. Eng. C.

[64] Delgado-Ruiz R., Romanos G. (2018). Potential causes of titanium particle and ion release in implant dentistry: A systematic review. Int. J. Mol. Sci..

[65] Asirvatham A., Devadoss D., Kujur A., Selvam A., Devi J.N., Mary S.J. (2024). Anti corrosion activity of CRF (Cardiac Risk Free) drug for SS316L, Ni–Ti, and Ti-6Al-4V in artificial blood plasma. Chem. Afr..

[66] Verdeguer P., Gil J., Punset M., Manero J.M., Nart J., Vilarrasa J., Ruperez E. (2022). Citric acid in the passivation of titanium dental implants: Corrosion resistance and bactericide behavior. Materials.

[67] Souza J.G., Cordeiro J.M., Lima C.V., Barão V.A. (2019). Citric acid reduces oral biofilm and influences the electrochemical behavior of titanium: An in situ and in vitro study. J. Periodontol..

[68] Manickam A., Srinivasan G., Murugan J., Sivakumar S., Mohan S. (2026). Corrosion mitigation strategies for 316L stainless steel in biomedical implants: Advances in materials and surface modifications. J. Bio-Tribo-Corros..

[69] Koike M., Fujii H. (2001). The corrosion resistance of pure titanium in organic acids. Biomaterials.

[70] Lucchetti M.C., Fratto G., Valeriani F., De Vittori E., Giampaoli S., Papetti P., Manzon L. (2015). Cobalt-chromium alloys in dentistry: An evaluation of metal ion release. J. Prosthet. Dent..

[71] Koike M., Fujii H. (2001). In vitro assessment of corrosive properties of titanium as a biomaterial. J. Oral. Rehabil..

[72] Fovet Y., Gal J.Y., Toumelin-Chemla F. (2001). Influence of pH and fluoride concentration on titanium passivating layer: Stability of titanium dioxide. Talanta.

[73] Borgioli F. (2023). The corrosion behavior in different environments of austenitic stainless steels subjected to thermochemical surface treatments at low temperatures: An overview. Metals.

[74] Sun H., Meng S., Chen J., Wan Q. (2023). Effects of hyperlipidemia on osseointegration of dental implants and its strategies. J. Funct. Biomater..

[75] Revathi A., Borrás A.D., Muñoz A.I., Richard C., Manivasagam G. (2017). Degradation mechanisms and future challenges of titanium and its alloys for dental implant applications in oral environment. Mater. Sci. Eng. C.

[76] Wu X., Tamimi F. (2018). Pharmacological risk assessment for dental implants. Mandibular Implant Prostheses: Guidelines for Edentulous Geriatric Populations.

[77] Pourshadloo M., Jameel M.F., Romero-Parra R.M., Yeslam H.E., Shafik S.S., Kareem A.K., Zabibah R.S., Sharifianjazi F., Bathaei M.S. (2023). Synthesis of TiO_2_/rGO composite coatings on titanium alloys with enhanced anticorrosion performance in palmitic acid-incorporated physiological solutions. Ceram. Int..

[78] Plyukhin D.V., Tseylikman V.E., Tseylikman O.B., Sinitskiy A.I. (2015). Features of free radical lipid peroxidation and serum proteins at dental implants and peri-implantitis. Kazan. Med. J..

[79] Wang Y.N., Liu S. (2025). Lipid droplet accumulation impairs osseointegration by disturbing the osteogenesis–osteoclasis balance on titanium implant surface in hyperlipidemia. BMC Oral. Health.

[80] Winnett B., Tenenbaum H.C., Ganss B., Jokstad A. (2016). Perioperative use of non-steroidal anti-inflammatory drugs might impair dental implant osseointegration. Clin. Oral. Implant. Res..

[81] Delcaru C., Alexandru I., Podgoreanu P., Grosu M., Stavropoulos E., Chifiriuc M.C., Lazar V. (2016). Microbial biofilms in urinary tract infections and prostatitis: Etiology, pathogenicity, and combating strategies. Pathogens.

[82] Fonseca C., Barbosa M.A. (2001). Corrosion behaviour of titanium in biofluids containing H_2_O_2_ studied by electrochemical impedance spectroscopy. Corros. Sci..

[83] Al-Hawary S.I.S., Habash R.T., Abosaooda M., Hjazi A., Saleh E.A.M., Hassan Z.F., Bathaei M.S. (2024). TiO_2_/PEG as smart anticorrosion and drug-eluting platforms in inflammatory conditions. Heliyon.

[84] Kubacki G.W., Gilbert J.L. (2018). The effect of the inflammatory species hypochlorous acid on the corrosion and surface damage of Ti-6Al-4V and CoCrMo alloys. J. Biomed. Mater. Res. Part A.

[85] Prestat M., Vucko F., Holzer L., Thierry D. (2021). Microstructural aspects of Ti6Al4V degradation in H_2_O_2_-containing phosphate buffered saline. Corros. Sci..

[86] Cheng X., Roscoe S.G. (2005). Corrosion behavior of titanium in the presence of calcium phosphate and serum proteins. Biomaterials.

[87] Talha M., Ma Y., Kumar P., Lin Y., Singh A. (2019). Role of protein adsorption in the biocorrosion of metallic implants—A review. Colloids Surf. B Biointerfaces.

[88] Mabilleau G., Bourdon S., Joly-Guillou M.L., Filmon R., Baslé M.F., Chappard D. (2006). Influence of fluoride, hydrogen peroxide and lactic acid on the corrosion resistance of commercially pure titanium. Acta Biomater..

[89] Benea L., Bounegru I., Axente E.R., Buruiană D. (2023). Susceptibility of 316L stainless steel structures to corrosion degradation in salivary solutions in the presence of lactic acid. J. Funct. Biomater..

